# MicroRNA-874 is downregulated in cervical cancer and inhibits cancer progression by directly targeting ETS1

**DOI:** 10.3892/or.2022.8380

**Published:** 2022-07-25

**Authors:** Haihong Liao, Yunfei Pan, Yuefen Pan, Junjun Shen, Quan Qi, Liping Zhong, Wenshu Han, Qi Wang, Yizhen Jiang

Oncol Rep 40: 2389–2398, 2018; DOI: 10.3892/or.2018.6624

Following the publication of this paper, it was drawn to the Editors' attention by a concerned reader that certain of the Transwell migration assay data shown in Fig. 3A were strikingly similar to data appearing in different form in other articles by different authors. Owing to the fact that the contentious data in the above article were already under consideration for publication prior to its submission to *Oncology Reports*, the Editor has decided that this paper should be retracted from the Journal. The authors were asked for an explanation to account for these concerns, but the Editorial Office did not receive a satisfactory reply. The Editor apologizes to the readership for any inconvenience caused.

